# High throughput compound screening in neuronal cells identifies statins as activators of ataxin 3 expression

**DOI:** 10.1038/s41598-023-41192-4

**Published:** 2023-09-09

**Authors:** Fabian Stahl, Ina Schmitt, Philip Denner, Laura de Boni, Ullrich Wüllner, Peter Breuer

**Affiliations:** 1 German Center for Neurodegenerative Diseases, DZNE, Venusberg-Campus 1, 53127 Bonn, NRW Germany; 2 Department of Neurology, University Hospital Bonn, Venusberg-Campus 1, 53127 Bonn, NRW Germany; 3 Institute of Aerospace Medicine, German Aerospace Center, Cologne, Germany

**Keywords:** Drug discovery, Molecular biology, Neuroscience

## Abstract

The spinocerebellar ataxias (SCA) comprise a group of inherited neurodegenerative diseases. SCA3 is the most common form, caused by the expansion of CAG repeats within the ataxin 3 (*ATXN3*) gene. The mutation results in the expression of an abnormal protein, containing long polyglutamine (polyQ) stretches. The polyQ stretch confers a toxic gain of function and leads to misfolding and aggregation of ATXN3 in neurons. Thus, modulators of *ATXN3* expression could potentially ameliorate the pathology in SCA3 patients. Therefore, we generated a CRISPR/Cas9 modified ATXN3-Exon4-Luciferase (ATXN3-LUC) genomic fusion- and control cell lines to perform a reporter cell line-based high-throughput screen comprising 2640 bioactive compounds, including the FDA approved drugs. We found no unequivocal inhibitors of, but identified statins as activators of the LUC signal in the ATXN3-LUC screening cell line. We further confirmed that Simvastatin treatment of wild type SK-N-SH cells increases *ATXN3* mRNA and protein levels which likely results from direct binding of the activated sterol regulatory element binding protein 1 (SREBP1) to the *ATXN3* promotor. Finally, we observed an increase of normal and expanded ATXN3 protein levels in a patient-derived cell line upon Simvastatin treatment, underscoring the potential medical relevance of our findings.

## Introduction

Machado Joseph Disease (MJD) or Spinocerebellar Ataxia 3 (SCA3) is a neurodegenerative disorder caused by an abnormal expansion of the polyglutamine (polyQ) tract of the ataxin-3 gene (*ATXN3*)^1,2^.

ATXN3 is a deubiquitinating enzyme (DUB), involved in protein homeostasis via the ubiquitin proteasome system (UPS) and endoplasmic reticulum associated degradation (ERAD) by interaction with VCP/p97^3^. In SCA3, the expanded polyQ tract within *ATXN3*, leads to misfolding, aggregation and accumulation of mutated (and the normal) ATXN3 protein in nuclear inclusions in neurons. The pathological accumulation of ATXN3 in turn impairs protein homeostasis within neurons^3,4^.

As a result of the neurodegenerative process, SCA3 patients are severely disabled and die prematurely. Thus, reducing the amount of the disease-causing ATXN3 protein constitutes a promising target for treatment approaches. In the past, several screens with different paradigms for ATXN3 modulation have been performed in the quest for novel targets for SCA3. Druggable genome-wide- and drug library screenings mainly concentrated on the reduction of stably overexpressed ATXN3(PolyQ)protein and improvement of the resultant toxicity^5–9^.

The antipsychotic drug Aripiprazole was identified as a potential ATXN3(PolyQ) reducing drug in a primary cell-based *ATXN3* overexpression model. Treatment of transgenic *ATXN3 Drosophila* flies and mice confirmed the beneficial effects by increasing longevity in the flies and decreasing aggregated ATXN3 protein in both SCA3 animal models^8^. More recently, Fardghassemi and colleagues found five neuroprotective drugs for ATXN3PolyQ induced toxicity in a compound screening assay performed in *C. elegans.* Thereby, they discovered that the transcription factor TFEB/HLH-30 was related to ATXN3 toxicity^9^. However, these approaches considering transgenic overexpression models of toxic ATXN3 alone missed modulators of endogenous ATXN3 regulation.

Here, we performed a reporter cell line-based high-throughput compound screening (HTS) assay to identify modulators of endogenous *ATXN3*. To our knowledge, this is the first study seeking for compounds to alter endogenous *ATXN3* expression levels. In our screening approach, we found no unequivocal inhibitors, but identified four statins i.e. Mevastatin, Atorvastatin, Fluvastatin and Simvastatin to increase LUC signal in the ATXN3-LUC screening cell line and further confirmed Simvastatin as an activator of *ATXN3* expression in SK-N-SH wild type cells. Statins are potent inhibitors of 3-hydroxy-3-methylglutaryl coenzyme A reductase (HMGCR). HMGCR is the rate-limiting enzyme in the mevalonate/cholesterol pathway. Inhibition of the enzyme results in cellular cholesterol reduction and in turn activates the sterol regulatory element binding proteins (SREBPs) which are key transcription factors for regulation of genes involved in cholesterol and lipid pathways. Three SREBPs are known and transcribed from two genes *SREBP1* and *SREBP2*. The *SREBP1* gene encodes for two SREBP1 isoforms (1a and 1c)^10,11^. In this study, we found that Simvastatin treatment increased ATXN3 levels by direct binding of the activated SREBP1 transcription factor to the *ATXN3* promotor in wild type SK-N-SH cells.

## Results

### Simvastatin leads to increased ATXN3 mRNA and protein levels by direct binding of activated SREBP1 transcription factor to the *ATXN3* promotor

**Figure 1:**
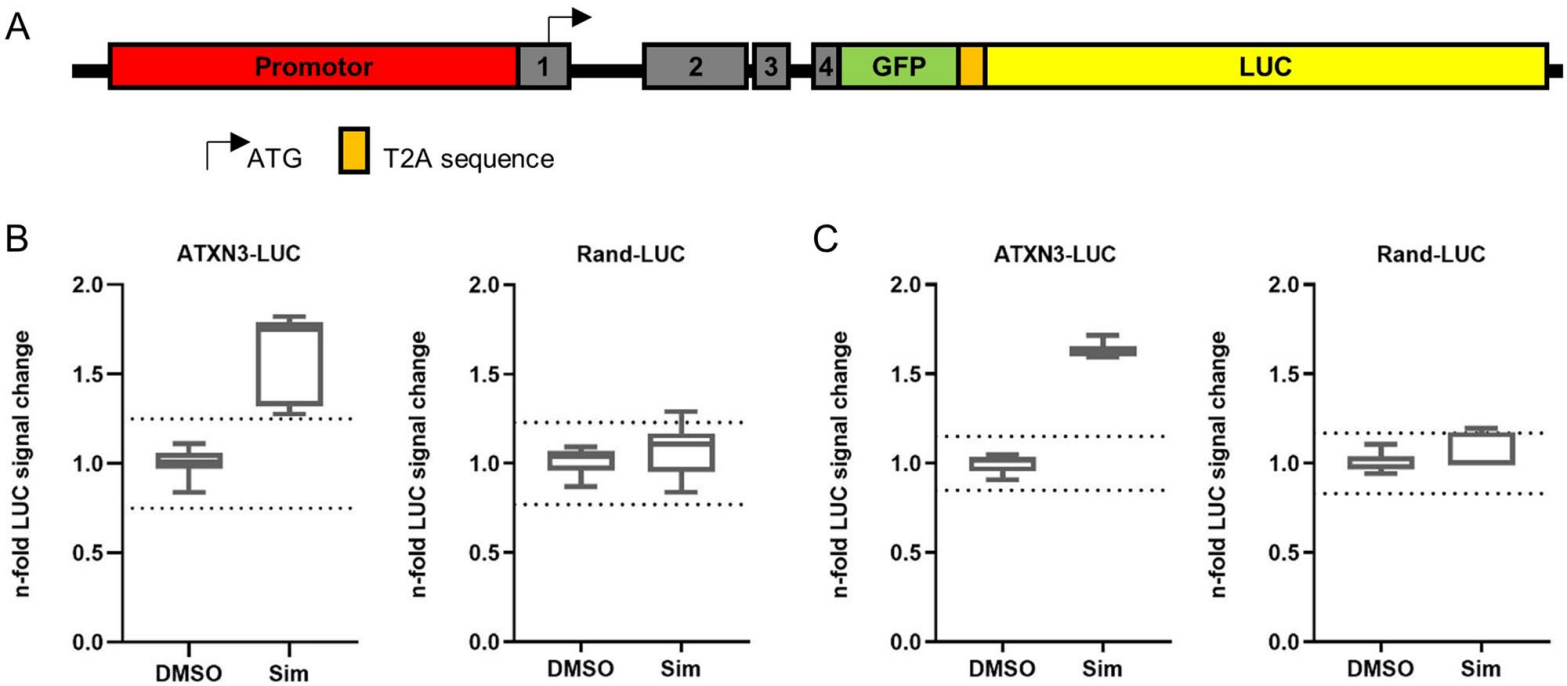
ATXN3-GFP-Luciferase (ATXN3-LUC) reporter cell line-based high throughput screening of 2,640 bioactive compounds, including FDA approved drugs identifies statins as modulators of ATXN3 expression. (**A**) Schematic overview of CRISPR/Cas9 generated, genomic ATXN3-LUC fusion under control of the endogenous *ATXN3* promotor. (**B**,**C**) Simvastatin (Sim) treatment increased LUC signal in ATXN3-LUC screening cell line but not in the control cell line, bearing a randomly integrated LUC cassette (Rand-LUC) after 8 h (**B**) and 24 h (**C**). Maximal n-fold change was observed after 8 h. Boxplot diagrams represents 5–95 percentile and dotted line in depicts the three-fold standard deviation (SD) from the mean of DMSO controls, (**B**) n = 7 and (**C**) n = 6 biological replicates. Sim treatment was applied at a final concentration of 10 µM. Additional information for ATXN3-LUC fusion see Supplementary Fig. S1.

We employed a similar HTS approach as published recently^12^. We used the CRISPR/Cas9 based gene editing to modify the endogenous *ATXN3* gene in a SK-N-SH wild type cell line by insertion of a GFP-T2A-LUC cassette (ATXN3-LUC) (Fig. 1A, Supplementary Fig. S1). A second SK-N-SH cell line with a random integration of the reporter cassette (Rand-LUC) served as a control for unspecific modulators and toxicity.

We found no inhibitors of *ATXN3*, but identified statins as activators of LUC signal in the ATXN3-LUC screening cell line (Fig. 1B,C, Supplementary Fig. S2–3). The compound library contained eight statins and two other HMGCR inhibiting drugs. Interestingly, only four statins i.e. Mevastatin, Atovarstatin, Fluvastatin and Simvastatin increased the LUC signal in the *ATXN3* SK-N-SH model, while Lovastatin, Pitavastatin, Pravastatin and Rosuvastatin showed no signal change compared to controls. Simvastatin showed the most consistent effect in the primary HTS and was thus chosen for further validation in non-modified SK-N-SH cells. The non-statin HMGCR inhibiting drugs, Clinofibrate and SR12813, were not effective in our screening assay.

**Figure 2:**
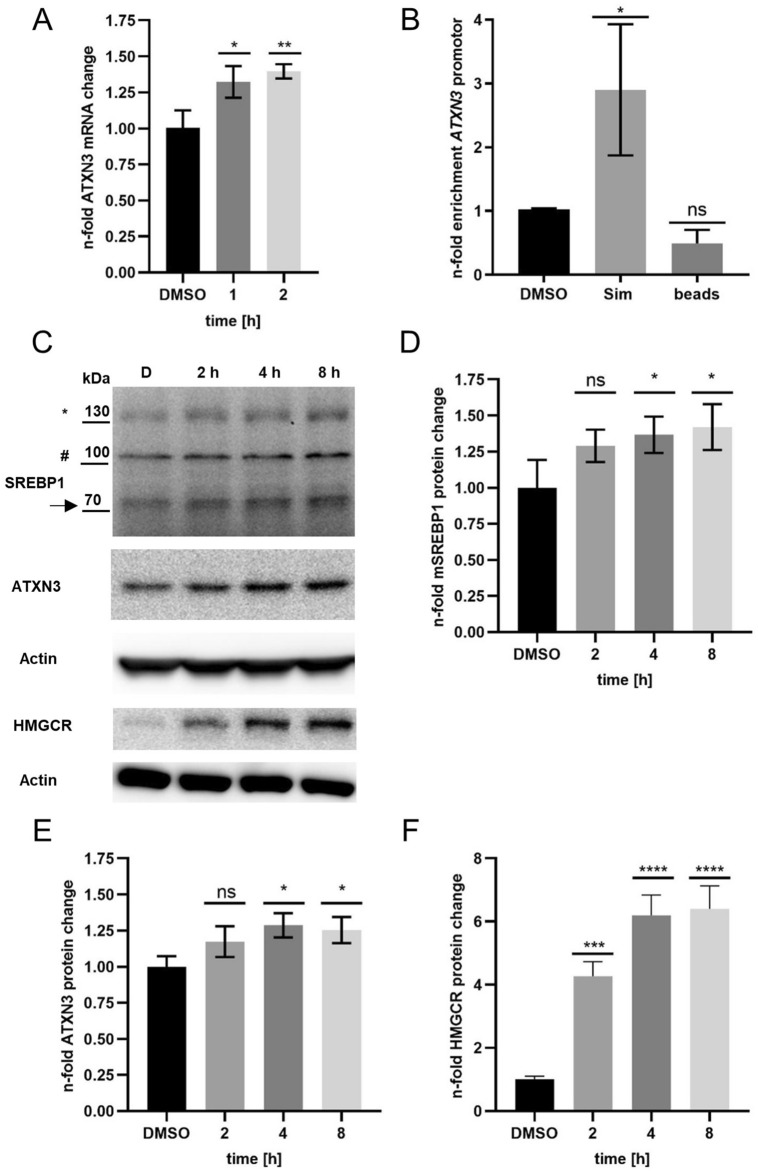
Simvastatin treatment increases *ATXN3* mRNA and protein levels, induces HMGCR protein level and activates the SREBP1 transcription factor and leads to binding of activated SREBP1 to the *ATXN3* promotor. (**A**) qRT-PCR of Simvastatin treated SK-N-SH wild type cells for 1–2 h. Data were normalized to three house—keeping genes and DMSO controls. (**B**) Relative n-fold enrichment of *ATXN3* promotor of three independent ChIP-qPCR experiments with the SREBP1 antibody (Proteintech 14088-1-AP) after 1 h Simvastatin (Sim) treatment. (**C**) Representative Western Blot (WB) of Sim treatment for 2–8 h. (**D**) Relative n-fold change of protein levels for mSREBP1, (**E**) ATXN3 and (**F**) HMGCR. Asterix and arrow indicate transcriptionally inactive- and mSREBP1, respectively. Hash: unspecific band. qRT-PCR Data were analysed using the ΔΔCT method by Pfaffl. ChIP-qPCR data were normalized to input and DMSO controls and represent the mean of three technical replicates for each experiment, respectively. WB data were normalized to actin and the mean of DMSO controls. Error bars indicate SD of three biological repetitions. For all experiments, a final concentration of 10 µM Sim was applied. Adjusted *p* values: *p < 0.05; **p = 0.005; ***p = 0.0002; ****p < 0.0001.

We performed time-course experiments in SK-N-SH wild type cells and Simvastatin was applied for 2, 4, and 8 h (h). The treatment showed a significant increase of *ATXN3* mRNA (1.4 fold after 2 h) and ATXN3 protein levels (1.3 fold after 4 h), compared to DMSO-treated control cells (Fig. 2A,C,E). Additionally, we observed increased levels of the activated sterol responsive element binding proteins (SREBP1) transcription factor and HMGCR (Fig. 2C,D,F). To corroborate the hypothesis that *ATXN3* mRNA and protein increase was due to direct binding of activated SREBP1 (mSREBP1) to the *ATXN3* promotor, chromatin immunoprecipitation (ChIP)—qPCR experiments for SREBP1 were performed and revealed an average *ATXN3* promotor enrichment of 2.9-fold after 1 h Simvastatin treatment, compared to DMSO-treated control cells (Fig. 2B) in wild type SK-N-SH.

### Time-controlled overexpression of active human SREBP1a in murine N2a cells increases endogenous ATXN3 protein levels

**Figure 3:**
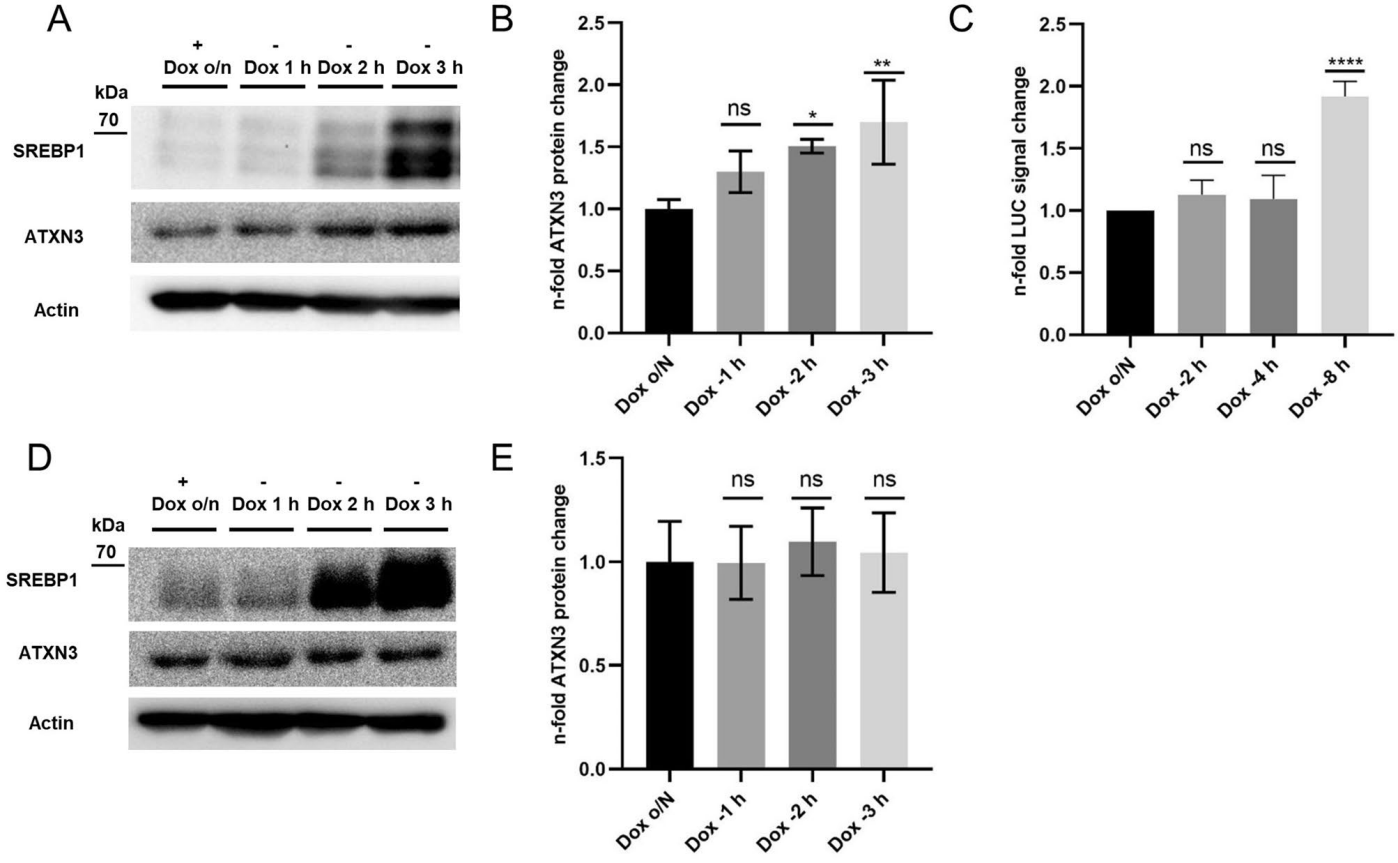
Overexpression of active human SREBP1a in murine N2a cells increases endogenous ATXN3 protein levels and the LUC signal of human *ATXN3* promotor containing LUC-reporter plasmid. Tet-Off murine N2a cells were transfected with pTREHyg-SREBP1a/c plasmids, respectively. Dox containing media were applied (ratio 1:1) to the transfection reaction after 4 h and incubated overnight. On the next day, protein expression was induced by Dox removal for 1–3 h (Dox − 1 to − 3 h) (**A**,**B**,**D**,**E**). Endogenous ATXN3 protein levels were normalized to actin and the mean of + Dox o/n. Three biological repetitions were performed. For the LUC-reporter assay, Dox was removed for 2–8 h and LUC signal was normalized to signal of co-transfected *Renilla* luciferase and Dox o/N. Three independent experiments were performed and were measured in technical triplicates. Data represent the mean of each triplicate. Error bars indicate the SD. Adjusted p values: *p < 0.05; **p < 0.01; ****p < 0.0001.

To assess, which SREBP1 isoform increases levels of ATXN3, we used a tetracycline-controlled trans-activator (tTA) containing (pTet-Off)- and doxycycline (Dox)-responsive murine N2a cell line. This system allows the transient and time-controlled overexpression of the constitutively active human SREBP1a- and 1c isoforms, which were previously cloned into pTREhyg plasmids. While human SREBP1 expression was completely restricted by overnight incubation with Dox, removal for 1–3 h enabled the expression of human SREBP1a- and 1c proteins. We observed significantly increased endogenous ATXN3 protein levels after two (1.5-fold) and three (1.7-fold) hours for human SREBP1a overexpression (Fig. 3A,B) while SREBP1c was inactive (Fig. 3D,E). To confirm SREBP1a binding to the human *ATXN3* promotor, we used the same expression system and co-transfected a LUC-reporter plasmid (pGL4 2.3) containing the promotor region of human *ATXN3* together with the human SREBP1a (pTREhyg)- and the *Renilla* luciferase plasmid for normalization. Cells were incubated overnight with Dox and removal for 2–8 h lead to significant increased LUC signal after 8 h (Fig. 3C).

### Simvastatin leads to increased ATXN3 and HMGCR protein levels in SCA3 patient-derived, induced pluripotent stem cell (iPSC)-derived long-term self-renewing neuroepithelial-like stem cells (MJD1 lt-NES cells)

**Figure 4:**
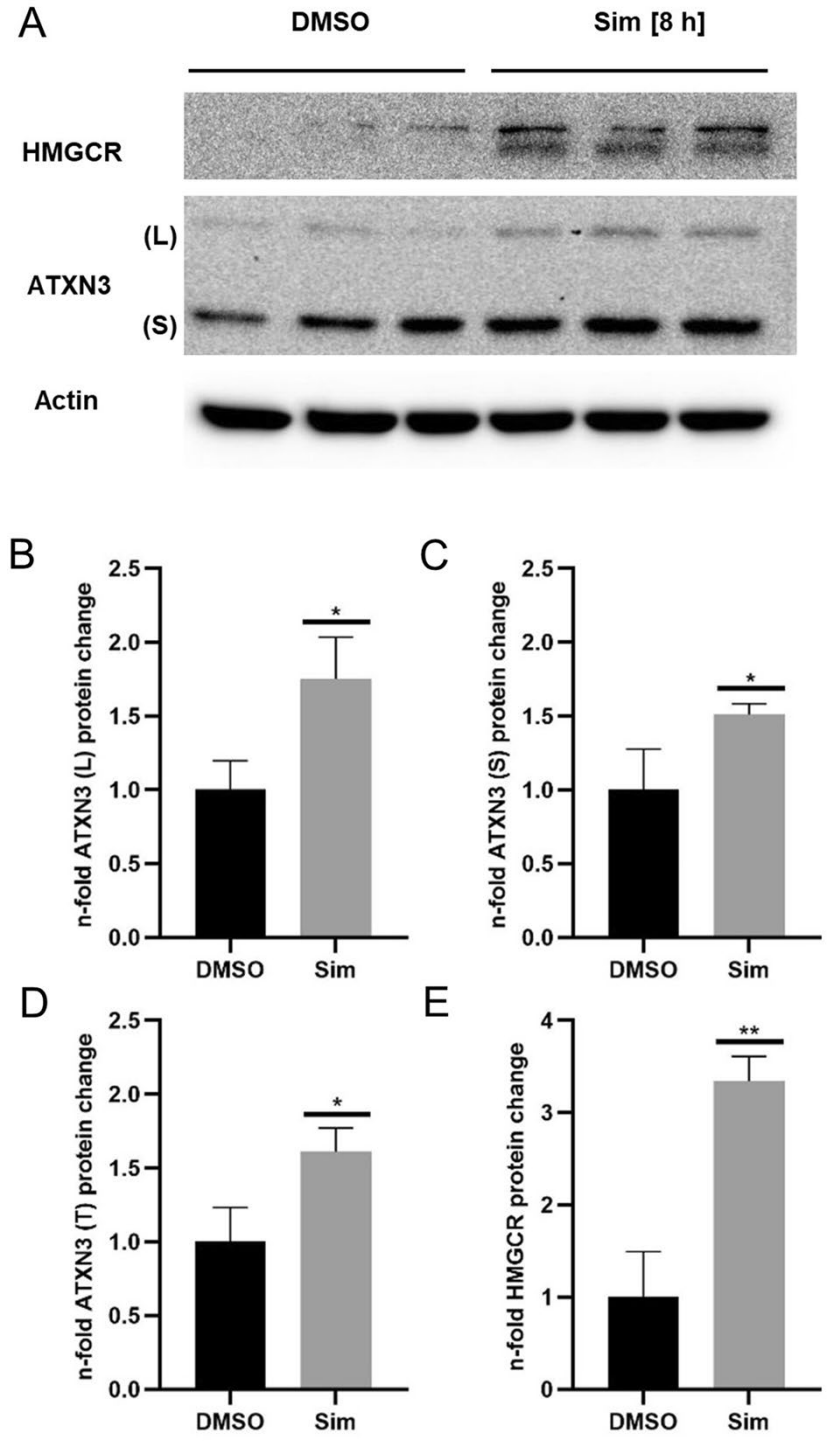
Simvastatin treatment increases ATXN3 and HMGCR protein levels in MJD1 lt-NES cells. (**A**) WB of Simvastatin (Sim) treated MJD1 lt-NES-cells. (**B**), (**C**) Quantification of the mutated ATXN3 polyQ allel (L) and the normal allel (S). (**D**) Summarized quantification of the L and S allels. (**E**) Quantification of HMGCR. Data represent biological triplicates and were normalized to actin and the mean of DMSO controls. Sim was applied for 8 h at a final concentration of 10 µM Sim. Adjusted *p* values: *p < 0.05; **p < 0.01. Several exposure images for Actin WB see Supplementary Fig. S6).

To find out whether Simvastatin treatment effects ATXN3 levels in a human model, we analysed SCA3 patient-derived MJD1 lt-NES cells. Simvastatin treatment led to similar and significantly increased levels of ATXN3 polyQ (L) (~ 1.75-fold)- and the normal allel (S) (~ 1.5-fold) which were quantified separately and summarized (T) (~ 1.6-fold), respectively (Fig. 4A–D). We also observed significantly increased HMGCR levels (~ 3.3-fold) (Fig. 4A,E).

## Discussion

Using a LUC reporter cell line-based HTS screening assay of 2640 bioactive substances, including FDA approved drugs we identified statins as activators of ATXN3 expression, while no inhibitors of *ATXN3* expression were found in this compound library.

The library contained eight different statins of which Mevastatin, Atovarstatin, Fluvastatin and Simvastatin were effective (Supplementary Fig. S3). Non-effective statins like Pravastatin and Rosuvastatin are considered as hydrophilic and may not easily pass cell membranes via diffusion. Thus, they may only reach low intracellular concentrations^16^. The other two statins, Lovastatin and Pitavastatin are lipophilic and should pass the cellular membranes but (also Prava- and Rosuvastatin) have also been shown to be less active in the reduction of total cholesterol levels in neuronal SK-N-MC cells compared to the four effective statins in our HTS. Furthermore, Simvastatin showed the highest reduction of total cholesterol in this assay, followed by Fluvastatin, Mevastatin and Atorvastatin^17^.

Simvastatin showed the most consistent effect in the primary HTS and led to increased *ATXN3* mRNA and protein levels in wild-type SK-N-SH cells (Fig. 2A,C,E). Mechanistically, this induction likely occurs by direct binding of the activated human SREBP1 to the *ATXN3* promotor as shown in ChIP-qPCR analyses in SK-N-SH wild type cells (Fig. 2B,C,D). In line with others, we observed increased protein levels of HMGCR after Simvastatin treatment in the SK-N-SH cells (Fig. 2F)^15^.

The human and murine *ATXN3* promotors contain putative SREBP1 binding motifs^18–22^ (Supplementary Figs. S4 and S5) and time-controlled overexpression of the active human SREBP1a in murine Neuro 2a (N2a) cells increased protein levels of ATXN3 (Fig. 3A,B). The same model system was used to confirm SREBP1a binding to the human *ATXN3* promotor by co-transfection of SREBP1a and a LUC-reporter plasmid containing the promotor sequence of human *ATXN3* (Fig. 3C), suggesting a similar SREBP1 dependent regulation of murine- and human ATXN3 levels.

To investigate whether Simvastatin modifies levels of ATXN3 polyQ protein in a human neuronal disease model, patient-derived MJD1 lt-NES cells were treated. As previously observed in the SK-N-SH cells, Simvastatin treatment led to increased levels of mutant and normal ATXN3-, and HMGCR protein (Fig. 4).

Statins are specific inhibitors of HMGCR, the rate-limiting enzyme in the mevalonate/cholesterol pathway^10^. Inhibition of HMGCR leads to reduced sterol levels in the cell, which in turn activates SREBPs to restore lipid and fatty acid homeostasis. SREBPs belong to the classic basic helix-loop-helix leucine zipper (bHLH zip) transcription factor family and consist of three proteins, SREBP1a, 1c and SREBP2 which are transcribed from two genes. SREBP1a and 1c differ in the length of their N-terminal transactivation domain and are involved in cholesterol- (SREBP1a) and fatty acid (SREBP1a/1c) homeostatic pathways. SREBP2 is encoded by the *SREBP2* gene and mainly involved in the cholesterol pathway^11,23^.

Cholesterol levels are sensed by the endoplasmic reticulum (ER) transmembrane-SREBP cleavage activating protein (SCAP) which is directly bound to SREBP. At low cholesterol levels, the SCAP-SREBP complex is transported to the Golgi via COPII vesicles and subsequent processing of SREBP by site 1 and 2 proteases leads to activation and nuclear translocation of the mature N-terminal (mSREBP) transcription factor. At high/normal cholesterol levels the SCAP-SREBP complex is retained in the ER membrane by direct interaction of SCAP with the insulin induced genes, INSIG1 and 2^13^.

The ability of statins to pass cellular membranes or the blood brain barrier (BBB) are determined by their physico-chemical properties. Highly lipophilic statins like Simvastatin and Lovastatin may easily cross via passive diffusion whereas less lipophilic statins including Fluvastatin and Pravastatin are discussed to be actively transported via organic anion transporter polypeptide 2 (OATP2) or monocarboxylic acid transporter (MCT)^24,25^. As a response to statin treatment, differential gene expression and slightly reduced cholesterol levels were observed in the brain, indicating potential regulatory properties in this tissue^26^. Thelen and colleagues^27^ found that short-term and high dose Simvastatin treatment in mice affected cholesterol synthesis and increased HMGCR mRNA levels in the brain, in contrast to pravastatin. In 2019, Fracassi and colleagues performed a broad meta study about the effect of statins in the brain e.g. mental disorders and neurodegenerative diseases like Alzheimer’s disease, Parkinson’s disease, and Huntington’s disease. They concluded, that more research is required as studies failed to confirm beneficial effects of statin treatment initially observed in neurodegenerative disease models^25^. Dependent on the pathophysiological condition of cholesterol levels in the respective neurodegenerative disease, in our opinion statin treatment requires careful consideration.

Little is known about altered cholesterol homeostasis in SCA3. Transcriptional changes of genes related to the cholesterol biosynthesis pathway in the brain of SCA3 transgenic mice have been reported^28^. Nobrega and Collegues (2019) recently found that cholesterol 24-hydroxylase (CYP46A1) is decreased in cerebellar extracts of SCA3 patients and SCA3 mice. CYP46A1 is the key enzyme allowing efflux of brain cholesterol via BBB into circulation and activating brain cholesterol turnover. Restoring CYP46A1 levels in SCA3 mice led to reduced ATXN3PolyQ accumulation and neuroprotection by alleviation of motor impairments. Vice versa, knocking-down of CYP46A1 in mice brain impairs cholesterol metabolism and resulted in severe neurodegeneration. The authors showed that CYP46A1 expression improved the endosomal–lysosomal pathway and increased the autophagy rate in general, linking a role of brain cholesterol pathway to mechanisms mediating clearance of aggregated proteins^29^.

**Figure 5:**
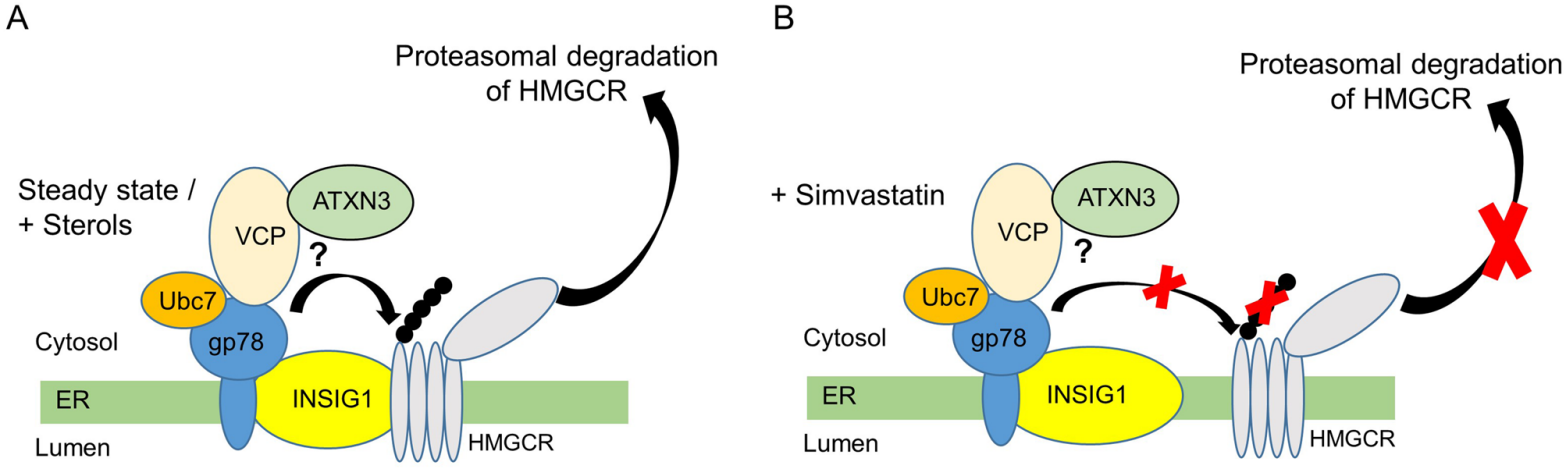
Putative role of ATXN3 in cholesterol homeostasis. Cholesterol homeostasis is tightly controlled at different levels within the cell. (**A**) Under normal (steady state) or under high sterol (+sterol) levels in a non-statin condition, ATXN3 may be involved in the sterol-induced and INSIG dependent, regulatory feedback degradation of HMGCR. (**B**) Statin treatment increases HMGCR levels by two mechanisms: First, it activates the SREBP pathway to upregulate genes involved in cholesterol homeostasis (including HMGCR, ATXN3). Second, statins strongly increase HMGCR protein levels by abolishing INSIG dependent ubiquitination and subsequent proteasomal degradation of the reductase. Modified from^13–15^. *ER* endoplasmic reticulum, *VCP* valosin-containing protein, *Ubc7* ubiquitin-conjugating enzyme E2 7, *gp78* glycoprotein78 E3 ubiquitin ligase, *INSIG1* insulin-induced gene 1, *HMGCR* 3-hydroxy-3-methylglutaryl coenzyme A reductase.

Although the above-mentioned studies observed dysregulation of genes within the cholesterol synthesis pathway or cholesterol turnover in SCA3 models, no data have yet described a distinct role of wild type or pathogenic ATXN3 in cholesterol homeostasis. We observed that Simvastatin treatment of patient-derived MJD1 lt-NES cells led to increased levels of mutant ATXN3 protein pointing to a possible implication of statin treatment in SCA3 patients (Fig. 4). Based on our work, we postulate a putative new role of ATXN3 in cholesterol homeostasis by functioning as a DUB enzyme acting in concert with the known interaction partner VCP/p97^30,31^ to stabilize or target key proteins of the cholesterol homeostasis for ERAD (Fig. 5)^13–15^, opening a novel door for future SCA3 research.

## Methods

### Cultivation and cell treatments of human neuroblastoma and N2A cell lines

The wild type human SK-N-SH neuroblastoma cell line was purchased from the European Collection of Authenticated Cell Cultures (ECACC) and used for the generation of our screening cell lines.

The wild type mouse Neuro 2A (N2a) was purchased from American Type Culture Collection (ATCC). All cell lines were cultivated in DMEM Glutamax (Gibco) supplemented with 1% penicillin/streptomycin (Gibco) and 10% inactivated FBS (Sigma-Aldrich), respectively. For detachment, cells were treated with 0.05% Trypsin–EDTA 1x (Gibco) for 10 min at 37 °C. All compounds were purchased from Selleckchem.

### Cultivation of SCA3 patient-derived, induced pluripotent stem cell (iPSC)-derived long-term self-renewing neuroepithelial-like stem cells (MJD1 lt-NES cells)^32^

Cells were plated on poly-l-ornithine/laminin (both Sigma) coated tissue culture plates and cultivated in a stock media (DMEMF12 Glutamax (Gibco) supplemented with 1% penicillin/streptomycin (Gibco), 1:100 N2 supplement (Gibco), 0.8 g D-glucose) with alternating concentrations of growth factors. On day one, cells were plated in stock media supplemented with 10 ng/ml EGF (Gibco) and 10 ng/ml FGF (Gibco). On the second day media was changed by stock media containing 40 ng/ml EGF and 40 ng/ml FGF. For further cultivation of MJD1 lt-NES cells, media was changed every day with alternating concentrations of growth factors (low: 10 ng/ml and high: 40 ng/ml). Accutase (Sigma) was diluted 1:3 with PBS and used for cell detachment (10 min room temperature). Treatments were performed in media with low growth factor concentration.

### Generation of *ATXN3*-*GFP*-*LUC* reporter- and control cell line via CRISPR/Cas9 gene editing and homologous recombination

CRISPR target sites for *ATXN3* exon 4 were selected from the web tool chopchop (https://chopchop.cbu.uib.no/)^33^ using the genomic sequence of *ATXN3* Exon4 (EU009923.1) and cloned into GeneArt CRISPR Nuclease Vector Kit (Thermo Fisher Scientific) according to manufacturer’s protocol. Primers for *ATXN3*_CRISPR target site 7 (TS7) Exon 4 were as follows: forward GATACTCTGGACTGTTGAAC**GTTTT**, reverse GTTCAACAGTCCAGAGTATC**CGGTG**.

For cloning of the homologous recombination (HR) vector HR150PA-1 (PrecisionX HR Targeting Vectors, System Bioscience), primers for the HR arms were tagged with 5′ palindromic sequences for respective restriction enzymes (bold). Amplification of HR arms were performed with Herculase II Fusion DNA Polymerase (Agilent) from genomic DNA (gDNA) of SK-N-SH. Primers were as follows: left HR arms upstream *GFP*-*LUC* cassette (HR150PA-1), forward **GAATTC**AAGCGGCAAGCCCATGACC and reverse **GAATTC**AGGATTAGTTCTAAACCCCAAACTTTC, ***EcoRI***; for right HR arm downstream *GFP*-*LUC* cassette (HR150PA-1), forward **GGATCC** AACAGTCCAGAGTATCAGAGGCTCAG and reverse **GTCGAC** GACAGGACCTCCCTTTGTTGCCC, ***BamHI*** and ***SalI***, respectively. Total length of HR arms was as follows: left HR arm 816 bp and right HR arm 702 bp. PCR products were sub-cloned into pJET1.2 (CloneJET PCR Cloning Kit, Thermo Fisher Scientific) and finally inserted into the HR150PA-1. Vector integrity was confirmed by sequencing. All restriction enzymes were fast digest enzymes and purchased from Fermentas, Thermo Fisher Scientific.

### Transfection, selection and screening of the *ATXN3*-*GFP*-*LUC* knock-in cell line

Performed as described in Stahl et al.^12^.

### Generation of stable tetracycline-controlled trans-activator (tTA) containing (pTet-Off)-N2a cell line

Wild type N2a cells were transfected with the pTet-Off regulatory plasmid according to manufacturer’s protocol (ClontechTet-Off Gene Expression System). Selection pressure was applied after 24 h and maintained for 1–2 weeks. Single colonies were picked and transferred into 96 well plates. To check for the best responding cell line, the single colonies were expanded in 24 well plates and transfected with the pTRE2hyg-LUC plasmid to measure luciferase activity in plus doxycycline (1:1000)—and minus doxycycline conditions. Doxycycline was purchased from Sigma.

### Cloning of SREBP constructs into pTRE2hyg plasmid

We purchased the pcDNA3.1-2xFLAG-SREBP1a, and 1c plasmids (#26801 and #26802) from addgene and cloned the coding sequence into pTRE2hyg vectors. pCDNA3.1 was linearized with XbaI and blunted. The SREBP sequences were finally excised with BamHI and cloned into BamHI and EcoRV digested pTRE2hyg plasmid (ClontechTet-Off Gene Expression System). pcDNA3.1-2xFLAG-SREBP1a, and 1c plasmids were a gift from Timothy Osborne (#26801 and #26802)^34^.

### Cloning of human *ATXN3* promotor into pGL4 2.3 plasmid

For cloning of the ATXN3 promotor into pGL 4 2.3 (Promega) plasmid, genomic DNA from SK-N-SH was amplified with forward **CTCGAG**ccttaacctctccgtgcc and reverse **AAGCTT**acgcagaccaatcaccc primer flanking the *ATXN3* promotor (promotor sequence see Supplementary Fig. S4) and sub-cloned in TOPO TA-vector (Thermofisher). The PCR product was excised with ***Xho*** and ***HindIII*** and cloned into ***Xho***, ***HindIII*** opened pGL 4 2.3. Vector integrity was confirmed by sequencing.

### Transfection of pTRE2Hyg plasmids into pTet-Off containing N2a cells

Transfection of N2A cells was performed with the Roti-Fect PLUS (Roth) transfection reagent according to manufacturer’s protocol. Cells were seeded in 12 well plates 4 h prior transfection. A total of 1 µg plasmid DNA was transfected to each well. The DNA to Roti-Fect ratio was 1:3 (1 µg and 3 µl). After 4 h, doxycycline-containing media (1:2 × 10^5^) was added to the transfection reaction (ratio 1:1) and incubated overnight. On the next day, doxycycline containing media was changed and protein expression was induced by doxycycline removal for 1–3 h.

### Isolation of nucleic acids

#### Genomic DNA (gDNA)

Performed as described in Stahl et al.^12^.

## PCR

### Standard PCR and gel-electrophoreses

For the generation of the homologous recombination arms 100–200 ng DNA was amplified in a total volume of 20 μl. Mastermix was prepared at final concentrations of 1 × reaction buffer (BioTherm, Genecraft), 250 µM dNTPs (Thermo Fisher Scientific), 0.2 µM of each primer, 1unit Taq DNA polymerase (BioTherm, Genecraft) and filled up to 20 µl with H_2_O. After initial denaturation at 94 °C for 3 min PCR were run for 30 cycles (denaturation 94 °C for 30 s, annealing for 30 s at respective temperature, extension 68 °C for 1 min). PCR products were amplified in the Biometra TADVANCED thermocycler and separated in 1% TBE agarose gel containing 2.5 × GelRed Nucleic Acid Gel Stain (Biotium) for visualization. For the validation of *ATXN3-GFP-LUC*-fusion, mRNA was converted into cDNA (QuantiTect Reverse Transcription Kit, Qiagen) and amplified by using hMJD-27 CGTGGGGGCCGTTGGCTCCAGACAAA and HR150*GFP* reverse TGTCACGATCAAAGGACTCTGG primer.

#### Miniprep/Maxipreparation

Performed as described in Stahl et al.^12^.

## RT-qPCR assays

### *ATXN3* mRNA

For hit validation SK-N-SH wildtype cells were treated at effective concentrations of 10 µM for 2 h in 24 well plate format and triplicates. Total RNA was extracted with the RNeasy Mini kit (Qiagen). RT-qPCR reactions were performed with the QuantiTect SYBR Green RT-PCR Kit (Qiagen) in a 96 well format and run in the Applied Biosystems HT7500 cycler. We used 75 ng of total RNA for amplification. Primers were as follows: *ATXN3*, forward CACGAGAAACAAGAAGGCTCAC and reverse CTCCTTCTGCCATTCTCATCC.

*ATXN3* mRNA expression were normalized to ubiquitin C (*UBC*), glucuronidase beta *GUSB* and hypoxanthine phosphoribosyl-transferase 1 (HPRT1) housekeeping genes as described in Stahl et al.^12^.

Relative mRNA levels were calculated using the ΔΔCT Method for multiple housekeeping genes from Pfaffl, published in “A–Z of quantitative PCR”^35^.

#### ChIP-qPCR

ChIP-qPCR was performed with Sigma Jumpstart SYBR Green Mastermix in a total volume of 25 µl. We used 3 µl of ChIP-DNA as a template. Primer were designed according to SREBP binding site annotation in UCSC human genome browser^20,21^. Primers as follows: *ATXN3* Promotor SREBP binding site FW CCAGGTGAGCGGTCCAGAC and RV GCAGACCAATCACCCGTGA. IP data were normalized to input and DMSO control according to Pfaffl. As a negative control we used empty controls (chromatin incubated with beads without antibodies).

#### *LUC* assay

Bioactive compound collections L1700 (Selleckchem) were randomly spotted at a concentration of 10 µM in three independent experiments.

The screening process was fully automated and performed at the Laboratory Automation Technologies (LAT), DZNE Bonn. For the luciferase assay 1.5 × 10^4^ cells/well in a volume of 30 µl were seeded into nunc white 384 well plates (Thermo Fisher Scientific). Cells attached and grew for approx. 18 h at 37 °C before treatment. The pre-spotted 384 well compound plates (100 nl/well) were diluted with 25 µl medium/well and shaken for 5 min with 1200 rpm at RT. Subsequently, 10 µl of the compound dilution were applied to 384 well cell plates, resulting in a final concentration of 10 µM and incubated for 24 h at 37 °C. Controls were distributed on the assay plate in a fixed layout for all three independent experiments. The tested drugs were randomly distributed for the three experiments to avoid well location dependent effects. Cells were lysed by adding 40 µl of ONE Glo (Lysis Buffer and Luciferase Substrate, Promega) to each well (on top of medium), incubated for 5 min while shaking at 1200 rpm and luciferase signal was measured with the Paradigm Reader at 1200 ms integration time.

For hit definition the LUC signal of treated cells was normalized to untreated controls per plate. Compounds showing an increased (activators) or decreased (inhibitors) LUC signal of more than the three-fold standard deviation (SD) of the median of untreated controls were considered as effective modulators. Repeated experiments were conducted in Nuncwhite 96 well plates and measured with the Centro LB 960 (Berthold Technologies) at 1200 ms integration time.

### Luciferase reporter gene assay of pGL4 2.3 Luciferase vector containing the human *ATXN3* promotor sequence and pTRE2HYG-SREBP1a co-transfection

For the reporter assay N2A cells were seeded in 12 well plates. Cells were transfected 4 h later with Roti-Fect PLUS transfection reagent according to manufacturer’s protocol with 500 ng DNA of the LUC reporter plasmid (pGL4 2.3, Promega) and the pTRE2Hyg-SREBP1a. The co-reporter *Renilla* Luciferase (pRL-CMV, Promega) was co-transfected at a concentration of 50 ng and was used for normalization. After 4 h, doxycycline-containing media (1:2 × 10^5^) was added to the transfection reaction (ratio 1:1) and incubated overnight. On the next day, doxycycline containing media was changed and SREBP1a expression was induced by doxycycline removal for 2–8 h. Cells were lysed with 400 µl 1 × passive lysis buffer (Promega). For the luciferase and *Renilla* luciferase assays 40 µl of the cell lysates were transferred to 96 well Nuncwhite plates. The same amount of luciferase- or *Renilla* luciferase substrate (substrate composition upon request) was added respectively, and measured with the Centro LB 960 (Berthold Technologies) at 1200 ms integration time.

### SDS PAGE and western blot analysis

For SDS-PAGE cells were harvested and lysed in RIPA buffer (50 mM TrisCl pH 7.5, 150 mM NaCl, 10 mM MgCl_2_, 0.5% Triton X 100, 1% SDS) supplemented with Halt Protease Inhibitor-Cocktail (1 × final concentration) (Thermo Fisher Scientific) and 0.5 µl/ml benzonase (Merck) for 30 min on ice. Lysates were mixed with 4 × Laemmli loading buffer (200 mM TrisCl pH 6.8, 8% SDS, 6% β-mercapto-ethanol, 33% glycerol, spatula tip bromophenol blue) to a final concentration of 1 × and boiled for 10 min at 95 °C. Samples were loaded onto 10% SDS-PAGE gels.

### Western blot

Proteins were blotted onto methanol activated polyvinylidene difluoride (PVDF) membrane (GE Healthcare, Amersham Hybond), blocked with 0.5% casein PBS (Roth) for 1 h and incubated with respective antibodies ATXN3 (1:1,000, #986) SREBP1 (1:1000, Proteintech 14088-1-AP), beta actin (1:10,000, A5441, Sigma-Aldrich) and HMGCR (1:4000, ABS229, Millipore) primary antibodies overnight at 4 °C. Secondary HRP conjugated anti mouse antibody (1:4000, P0447, Dako) was applied for 1 h at room temperature. Secondary HRP conjugated anti rabbit antibody (1:4000, 7074 V, Cell Signaling Technology) was applied.

Membranes were washed three times with 1xPBS-TWEEN 20 0.1% (PBST 0.1%) and imaged with enhanced chemiluminescence (ECL) in the ChemoCam imager (Intas). Signals were quantified with the ImageJ software^36^. Treated cells were normalized to actin and DMSO controls, respectively.

### Chromatin immunoprecipitation (ChIP)

For ChIP assays, cells were plated in 10 cm dishes and treated the next day with DMSO or 10 µM Simvastatin in a total volume of 10 ml for 1 h. Cells were crosslinked with a final concentration of 0.85% formaldehyde (37% Stock, Sigma). The reaction was stopped after 8 min by adding glycine to a final concentration of 0.125 M. Cells were washed twice with ice cold PBS, scraped from plates and transferred to 15 ml falcon tubes. After centrifugation, the supernatant was removed and 1 ml of cell lysis buffer (10 mM Tris Cl pH 8, 10 mM NaCl, 0.2% NP40 and freshly added protease inhibitor) was added. Cells were lysed on ice for 10 min and centrifuged. Centrifugations were performed at 4 °C for 10 min at 1200 rcf. Supernatant was removed and 0.5 ml nuclei lysis buffer (50 mM Tris Cl pH 8, 10 mM EDTA, 1% SDS and freshly added protease inhibitor) was applied and incubated for 10 min at 4 °C. Samples were diluted with 0.5 ml RIPA buffer (without benzonase and SDS) and sonicated for 10 s in 10 intervals at cycle 2 and 40% power for three rounds with the Bandelin Sonopuls, HD2070, SH70G, type MS72. Chromatin was cleared by centrifugation at 4 °C for 10 min at 13,000 rcf and supernatant was transferred to fresh reaction tubes. To check fragment size, 50 µl of the samples were incubated with 5 µl of 5 M NaCl, 10 mg/ml RNAse (1 h, 37 °C) and 20 mg/ml proteinase K (3 h—o/N, 65 °C). Subsequently, agarose gel electrophoresis was performed to check chromatin size which is best between 200 and 700 bp length.

For ChIP assays, 400 µl of chromatin was diluted with 1 ml RIPA buffer (without benzonase and SDS). We used 2% of the chromatin as input control and added 7 µl (5.25 µg) of SREBP 1 antibody (Proteintech, 14,088–1-AP) for overnight incubation.

The next day, 20 µl Magna ChIP Protein A + G Magnetic Beads (Merck Millipore) were used for the pulldowns or empty controls (beads only) for 1.5 h at 4 °C. Samples were washed with 500 µl of low salt- (0.1% SDS, 1% Triton X-100, 2 mM EDTA, 20 mM Tris HCl pH 8, 150 mM NaCl), high salt- (equal to low salt, except 500 mM NaCl), lithium salt buffer (0.25 M LiCl, 1% NP40, 1% Sodium Deoxycholate, 1 mM EDTA, 10 mM Tris HCl pH 8) and PBS, respectively. Antibody-antigen complexes were eluted by incubation with 200 µl freshly prepared elution buffer (100 mM NaHCO_3_ and 1% SDS) for 15 min. Input samples were also incubated with 200 µl elution buffer. All samples were incubated with 10 µl 5 M NaCl, 10 mg/ml RNAse (1 h, 37 °C) and 20 mg/ml proteinase K (3 h—o/N, 65 °C) and purified with ChIP-DNA purification kit from Zymo.

### Statistics

We used one-way ANOVA (α = 0.05) followed by the recommended Dunnett’s multiple comparison test to check for statistical significance. Significance in Fig. 4B–E, was checked by the recommended unpaired- and two-tailed t-test. All statistical analyses were performed in GraphPad Prism 8.3.0.

### Supplementary Information

Supplementary Figures.

## Data Availability

The datasets used and analysed during the current study are available from the corresponding author on reasonable request.
