# Supplementary material for: High throughput compound screening in neuronal cells identifies statins as activators of ataxin 3 expression
Source: Sci Rep. 2023 Sep 9;13:14911. doi: 10.1038/s41598-023-41192-4 (PMC10492798; doi:10.1038/s41598-023-41192-4)
Supplement: Supplementary file 1 — Supplementary Figures. [file 41598_2023_41192_MOESM1_ESM.pdf]

High Throughput Compound Screening in Neuronal Cells Identifies Statins as Activators of Ataxin 3 Expression

Fabian Stahl[1,2], Ina Schmitt[2], Philip Denner[1], Laura de Boni[2,3], Ullrich Wüllner[1,2,4*], Peter Breuer[2,4*]

[1]DZNE, German Center for Neurodegenerative Diseases, Germany
[2]University Hospital Bonn, Department of Neurology, 53105, Bonn, NRW, Germany
[3]Institute of Aerospace Medicine, German Aerospace Center, Cologne, Germany

Corresponding authors:

PD Dr. rer. nat. Peter Breuer

University Hospital Bonn, Department of Neurology

Venusberg-Campus 1

53105 Bonn, NRW, Germany

Tel: +49 228 287 11569

Email: peter.breuer@ukbonn.de

Prof. Dr. Ullrich Wüllner

DZNE & University Hospital Bonn, Department of Neurology

Venusberg-Campus 1

53105 Bonn, NRW, Germany

Tel (DZNE): +49 228 287 15712

Tel (University Hospital Bonn, Department of Neurology): +49 228 287 15736

Email: ullrich.wuellner@dzne.de

* corresponding authors

[4] shared last authorship

Supplementary Figure S1

A

CGTGGGGGGGCCCGTGGCTCCAGACAAATAAACATGGAGTCCATCTTCCACGAGAAACAAGAAGGCT
CACTTTGTGCTCAACATTGCCTGAATAACTTATTGCAAGGAGAATATTTTAGCCCTGTGGAATTATCCT
CAATTGCACATCAGCTGGATGAGGAGGAGAGGATGAGAATGGCAGAAGGAGGAGTTACTAGTGAAGA
TTATCGCACGTTTTTACAGCAGCCTTCTGGAAATATGGATGACAGTGGTTTTTCTCTATTCAGGTTAT
AAGCAATGCCTTGAAAGTTTGGGGTTTAGAACTAATCCTGGAATTCATGCCCGCCATGAAGATCGAGT
GCCGCATCACCGGCACCCTGAACGGCGTGGAGTTCGAGCTGGTGGGCGGCGGAGAGGGCACCCCC
GAGCAGGGCCGCATGACCAACAAGATGAAGAGCACCAAAGGCGCCCTGACCTTCAGCCCCTACCTG
CTGAGCCACGTGATGGGCTACGGCTTCTACCACTTCGGCACCTACCCCAGCGGCTACGAGAACCCCT
TCCTGGCACGCC

B

MESIFHEKQEGSLCAQHCLNNLLQGEYFSPVELSSIAHQLDEEERMRMAEGGVTSEDYRTFL
QQPSGNMDDSGFFSIQVISNALKVWGLELILEFMPAMKIECRITGTLNGVEFELVGGGEGTPE
QGRMTNKMKSTKGALTFSPYLLSHVMGYGFYHFGTYPSGYENPFLAR

C

MESIFHEKQEGSLCAQHCLNNLLQGEYFSPVELSSIAHQLDEEERMRMAEGGVTSEDYRTFL
QQPSGNMDDSGFFSIQVISNALKVWGLELILFNSPEYQRLRIDPINERSFICNYKEHWFTVRKL
GKQWFNLNSLLTGPELISDTYLALFLAQLQQEGYSIFVVKGDLPDCEADQLLQMIRVQQMHRP
KLIGEELAQLKEQRVHKTDLERVLEANDGSGMLDEDEEDLQRALALSRQEIDMEDEEADLRRA
IQLSMQGSSRNISQDMTQTSGTNLTSEELRKRREAYFEKQQQKQQQQQQQQQQGDLSGQS
SHPCERPATSSGALGSDLGDAMSEEDMLQAAVTMSLETVRNDLKTEGKK

D

MPAMKIECRITGTLNGVEFELVGGGEGTPEQGRMTNKMKSTKGALTFSPYLLSHVMGYGFY
HFGTYPSGYENPFL […]

Supplementary Figure S1. Confirmation of in-frame ATXN3-Exon4-GFP-luciferase (LUC) genomic fusion. A) cDNA sequence of *ATXN3*-Exon4-*GFP-LUC* genomic fusion. To confirm the in-frame insertion of *GFP-T2A-LUC* into *ATXN3* Exon4, mRNA of the screening cell line was extracted, transcribed into cDNA and amplified with primers as follows: MJD-27 (upstream start codon *ATXN3*) CGTGGGGGCCGTTGGCTCCAGACAAA and HR150 GFP RV (within the *GFP* cassette) TTCTCGATGCGGGTGTTGG. B) *In-silico* translation of the sequenced cDNA (A) via expasy translater (https://web.expasy.org/translate/). C) Sequence was compared to uniprot ATXN3 protein sequence (https://www.uniprot.org/uniprotkb/P54252/entry) and D) to *in-silico* translated GFP sequence from the HR150 plasmid. Light grey underlined: start codon *ATXN3*, grey: coding sequence (CDS) *ATXN3* Exon1 to half of Exon4; red: EcoRI site; turquoise underlined: start codon *GFP* cassette; green: *GFP* sequence (partial).

Supplementary Figure S2

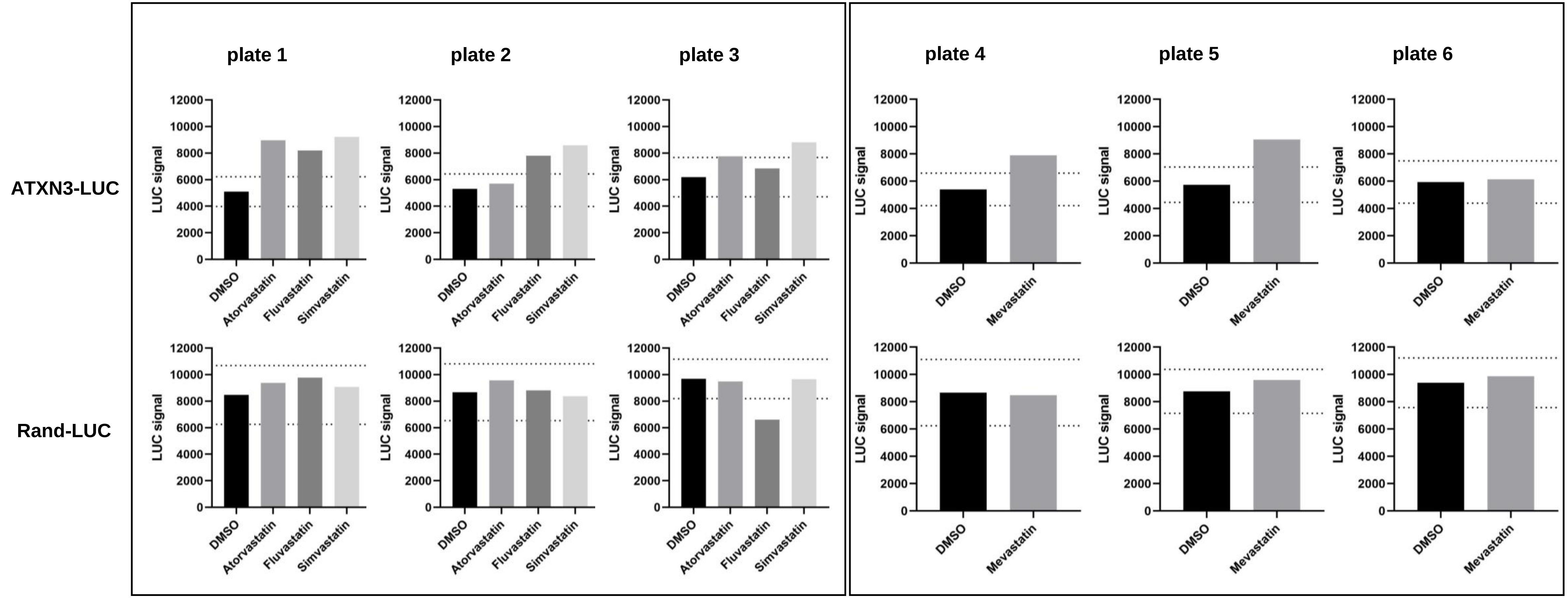

Supplementary Figure S2. Luciferase signal data (LUC signal) from the primary luciferase assay in the screening (ATXN3-LUC) and control cell line (Rand-LUC). Each compound was measured in biological triplicates. To exclude plate specific effects, all compounds were spotted in three independent 384-well plates, respectively. The dotted lines represent the three-fold standard deviation (3x SD) of the median of DMSO controls per plate. Compounds that increased LUC signal above 3x SD were considered as effective. Efficacy 0 = no effect observed; 1 = in 1/3 plates effective; 2 = 2/3 and 3 = 3/3 effective. LUC signals were represented as absolute values. Atorvastatin, Fluvastatin and Simvastatin were spotted on the same plate. DMSO controls per plate n= 58. Summary of efficacy see in Supplementary Figure (S3).

Supplementary Figure S3

Efficacy	Name	Solubility	Trade name
3	Simvastatin	Lipophilic	Zocor
2	Fluvastatin	Lipophilic	Lescol
2	Atorvastatin	Lipophilic	Lipitor
2	Mevastatin	Lipophilic	%
0	Lovastatin	Lipophilic	Mevacor/Altoprev
0	Pitavastatin	Lipophilic	Livalo
0	Pravastatin	Hydrophilic	Pravachol
0	Rosuvastatin	Hydrophilic	Crestor

Supplementary Figure S3. ATXN3-GFP-LUC reporter cell line-based high throughput screening of 2,640 bioactive compounds, including FDA approved drugs identifies statins as modulators of ATXN3 expression. List of Statins included in the screening library and sorted according to their efficacy. Initial screening was performed in three rounds of single treatments normalized to controls of respective plates (Supplementary Figures S2 A, B). Efficacy of 3 represents LUC signal increase above a threshold of the 3xSD from controls in 3/3 experiments. Efficacy 0 = no effect observed.

Supplementary Figure S4

A

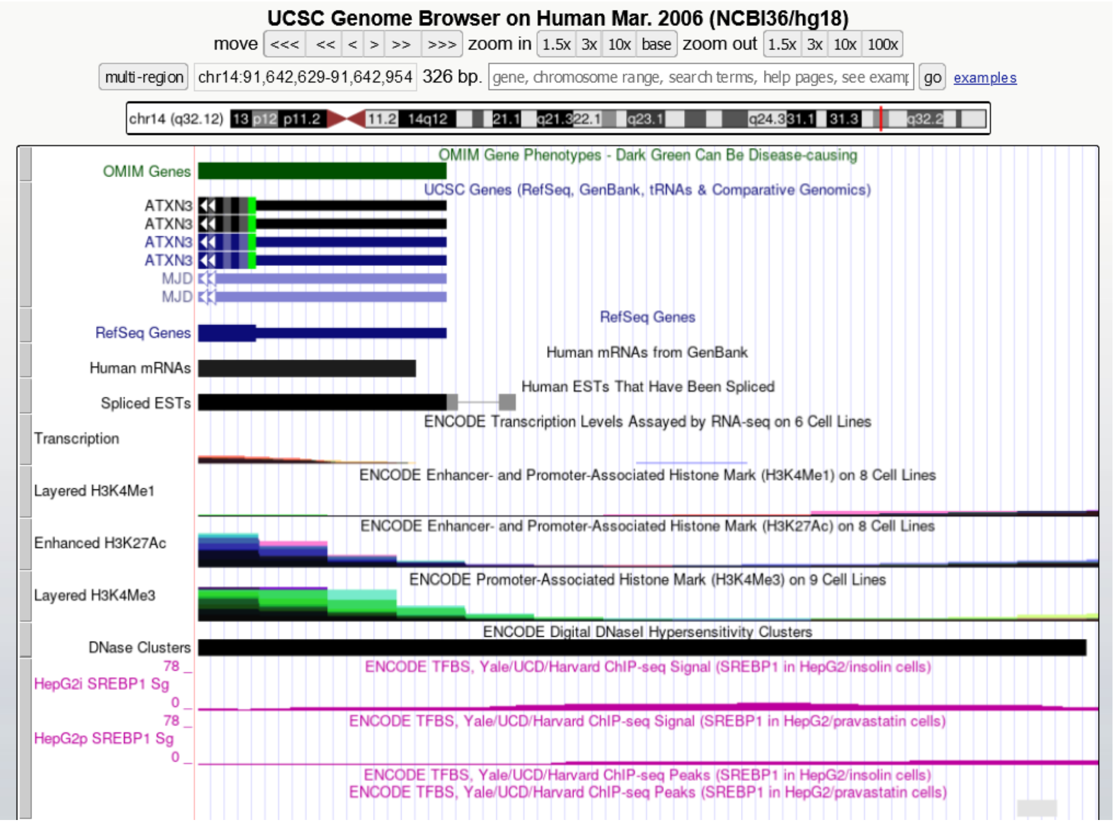

B

ENCODE TFBS, Yale/UCD/Harvard ChIP-seq Signal (SREBP1 in HepG2/pravastatin cells).

CTCGTGGAAGATGGACTCCATGTTTATTTGTCTGGAGCCAACGGCCCCCACGCCGAAC
CACCCCCTCCAGCTCCGCCCCCTGCCCCTCTCCTCCGCCCACCCCGCCCCTCCCCGG
CCCGCCCCGGTACCCCGCCCACGTGCCGCACGCAGACCAATCACCCGTGAGGATGC
GAGTGTCGTGAAGCGAAGAGCGAGTGAACGCCGGGGACACGTGACCTTGGTAGCGC
GTCCTCCTAGCTTCTGTTAGCGGGCCTTCTGGGGGATGCTACATCGCCACCTGCTGTC
TGGAGGATCTTCTCCGCTTCCCACCCATTTCTATGGATTCT

C

ATXN3 Promotor (NCBI: NG_008198.2).

CCTTAACCTCTCCGTGCCTCGGTTTCCTCATGTGTATGAATAACATCAACACCTACATCAAAGTTT
GCTGTATTAAATTTGATAATATATGCAAAGCATTTAGAAAAGTGCCTAGCTCATAGAAAGCCTTAT
GTAAATATTAACTATCATTTTTTTTCTTTTTTGGGGTGGTGGGGGAGGGGTTTCGCTTTTGTTGCC
CAGGCTGGCGTGCAATGGCACGATCTCGGCTCACCACAACCTCGGCCTCCCGGGTTCAAGCGA
TTCTCCTGCCTCAGCCTCCCGAGTACCTGGGATTACAGGCATGCACCACCACGCCCGGGTAATT
TTGTATTTTTAGTGGAGACGGGGTTTCTCCATGTTGGTCAGGCTGATCTCAAACTCCCGACCTCA
GGTGATCCGCCCGCCTCGGCCTCCCAAAGTGCTGGGATTACAAGCGTGAAGCACCTCGCCCGG
CCTAGCTATCATTTTTATACAAGTGCTGGGTTTTGGGAGAATGTAATGATGGCTTTTTTCTTACTAA
ACTTTCAGTGCAGGAGGAGGAGAAAGAAAGTAAATAGTTATATGAACACAGTAGAAAGTCAAAGT
GGAAAACAAAAAGAACATAGAACCCAGGTGAGCGGTCCAGACCTCCCCCCAGAAACCTAAGAAT
CCATAGAAATGGGTGGGAAGCGGAGAAGATCCTCCAGACAGCAGGTGGCGATGTAGCATCCCC
CAGAAGGCCCGCTAACAGAAGCTAGGAGGACGCGCTACCAAGGTCACGTGTCCCCGGCGTTCA
CTCGCTCTTCGCTTCACGACACTCGCATCCTCACGGGTGATTGGTCTGCGTGCGGCACGTGGGC
GGGGTACCGGGGCGGGCCGGGGAGGGGCGGGGTGGGCGGAGGAGAGGGGCAGGGGGCGGA
GCTGGAGGGGGTGGTTCGGCGTGGGGGCCGTTGGCTCCAGACAAATAAACATGGAGTCCATCT
TCCACGAGAAA

Supplementary Figure 4. Detailed information about retrieving SREBP1 binding motif at the *ATXN3* promotor. We used the motifmap database (https://motifmap.ics.uci.edu/) to find putative binding of SREBP1 to *ATXN3* promotor by using the "gene search" function. We selected the species alignment "Human (hg18 multiz28way_placental) and searched for *ATXN3* gene (NM_001127696). We screened the transcription factor (TF) entry list for the *ATXN3* promotor and found SREBP1. Further data were retrieved from the UCSC genome browser via a direct link from motifmap (by choosing SREBP1). A) Screenshot from UCSC genome browser shows SREBP1 Chromatin immunoprecipitation (ChIP)-sequencing (seq) signal data, generated by ENCODE TFBS, Yale/UCD/Harvard, at the *ATXN3* promotor (http://genome.ucsc.edu/cgi-bin/hgTracks?db=hg18&lastVirtModeType=default&lastVirtModeExtraState=&virtModeType=default&virtMode=0&nonVirtPosition=&position=chr14%3A91642596%2D91643084&hgsid=1662301856_SE1yh97ifny0lfha2aJvwt1dE5a3). B) The DNA sequence of Chlp-seq data were obtained by selecting SREBP1 (http://genome.ucsc.edu/cgibin/hgc?hgsid=1662301856_SE1yh97ifny0lfha2aJvwt1dE5a3&g=htcGetDna2&table=&i=mixed&l=91642628&r=91642954&getDnaPos=chr14%3A91%2C642%2C62991%2C642%2C954&db=hg18&hgSeq.cdsExon=1&hgSeq.padding5=0&hgSeq.padding3=0&hgSeq.casing=upper&boolshad.hgSeq.maskRepeats=0&hgSeq.repMasking=lower&boolshad.hgSeq.revComp=0&submit=get+DNA). C) Putative *ATXN3* promotor sequence. Red: promotor; grey: *ATXN3* exon1; light grey: *ATXN3* start codon; pink: start and end of ChIP-seq data alignment; yellow: qPCR primer for ChIP-qPCR experiments to amplify a part the *ATXN3* promotor region of SREBP1 ChIP-seq data.

Supplementary Figure S5

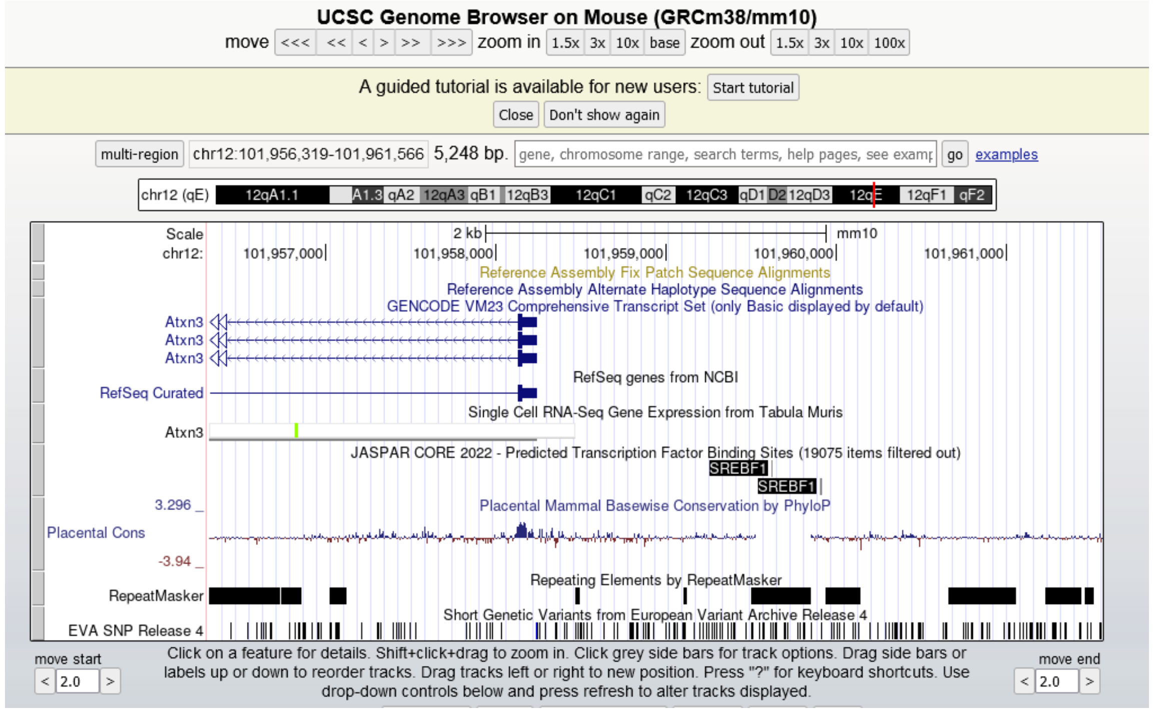

Supplementary Figure S5. Screenshot of the UCSC genome browser on Mouse. Predicted SREBF1 binding sites in murine *ATXN3* promotor by JASPAR CORE 2022 (https://genome.ucsc.edu/cgi-bin/hgTracks?db=mm10&lastVirtModeType=default&lastVirtModeExtraState=&virtModeType=default&virtMode=0&nonVirtPosition=&position=chr12%3A101956319%2D101961566&hgsid=1665753116_8Akmet9F5RUbYTMomIMNQa7jijRd).

Supplementary Figure S6

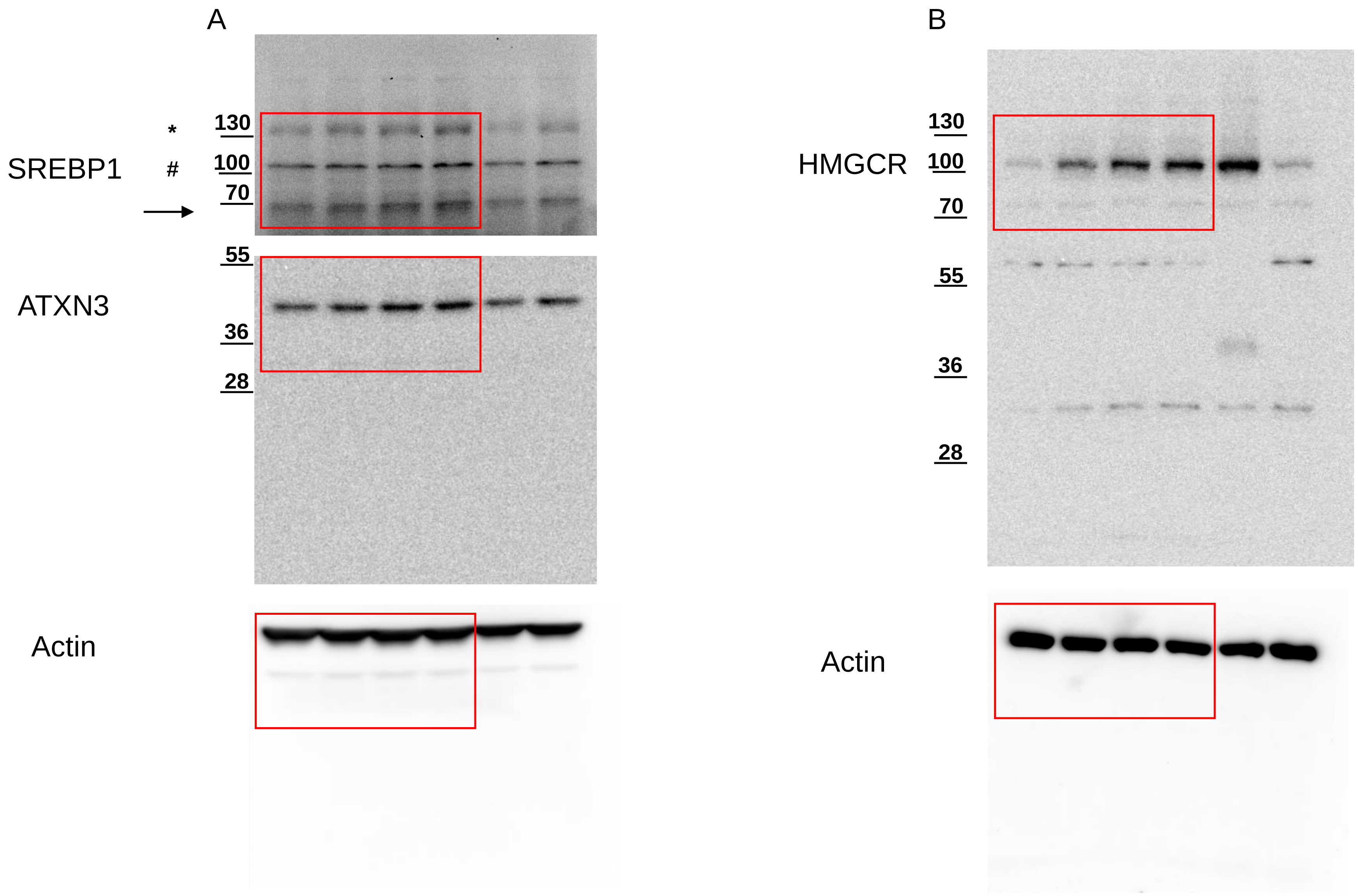

Supplementary Figure S6. Full-size Western Blot of Figure 2 C. A) Membrane was cut at 55 kDa before incubation with the indicated antibodies. Asterix: unprocessed SREBP1, hash: unspecific band, arrow: activated SREBP1 (mSREBP1). B) HMGCR and Actin Western Blot. Membrane was cut at 55 kDa after HMGCR development. The lower part was incubated with Actin antibody. Red boxed lanes were used for Figure 2 C.

Supplementary Figure S7

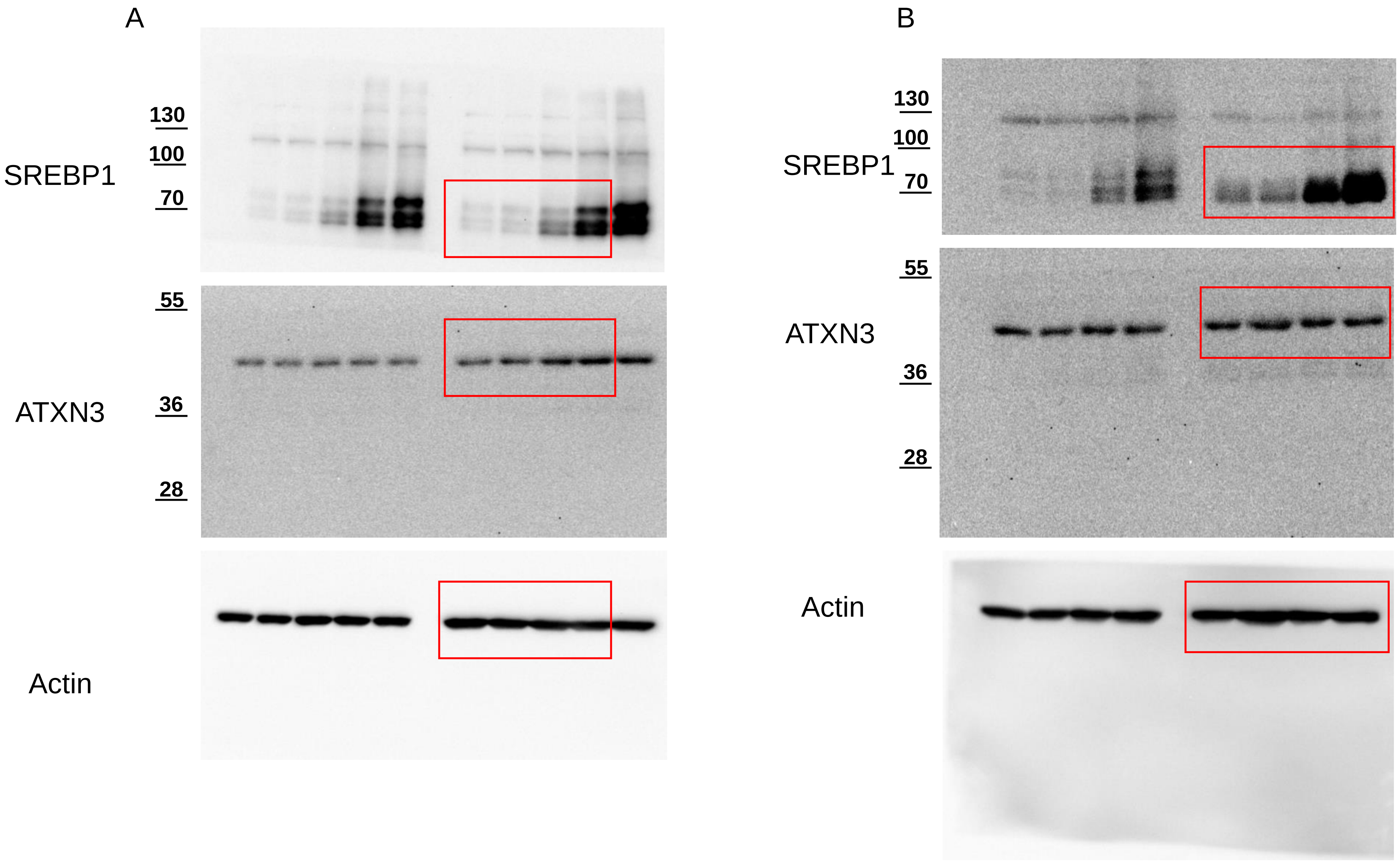

Supplementary Figure S7. Full-size Western Blot of Figure 3. Membranes were cut at 55 kDa to incubate with the indicated antibodies. A) Overexpressed SREBP1a (red boxed lanes) and B) SREBP1c (red boxed lanes) were shown in Fig. 3 A and B, respectively. Red boxed lanes were used for Figure 3.

Supplementary Figure S8

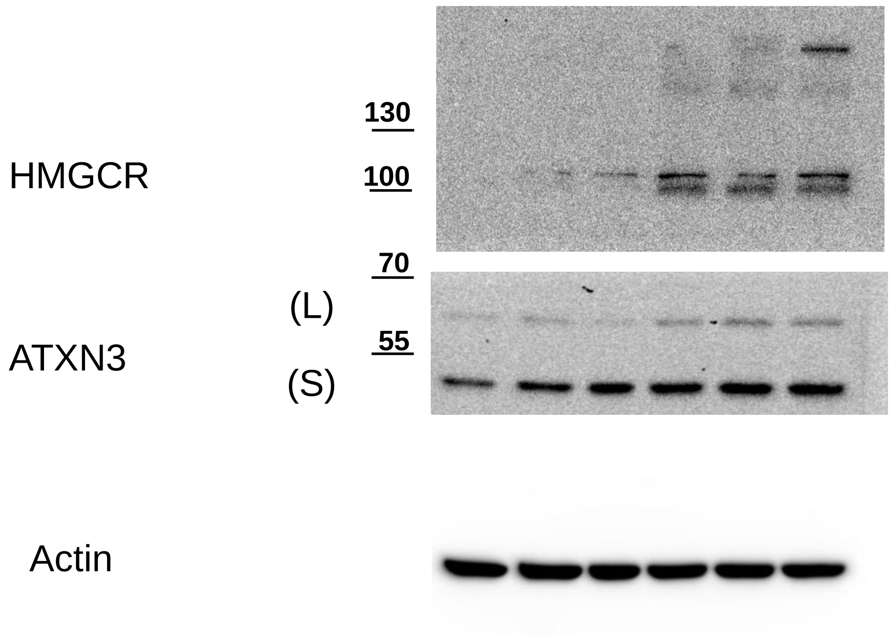

Supplementary Figure S8. Full-size Western Blot of Figure 4. Membrane was cut at ~70 kDa to incubate with the HMGCR, ATXN3 and Actin, respectively. L) Long ATXN3 allel and S) short allel. Several exposure images of Actin Western Blot see Supplementary Figure S9.

Supplementary Figure S9

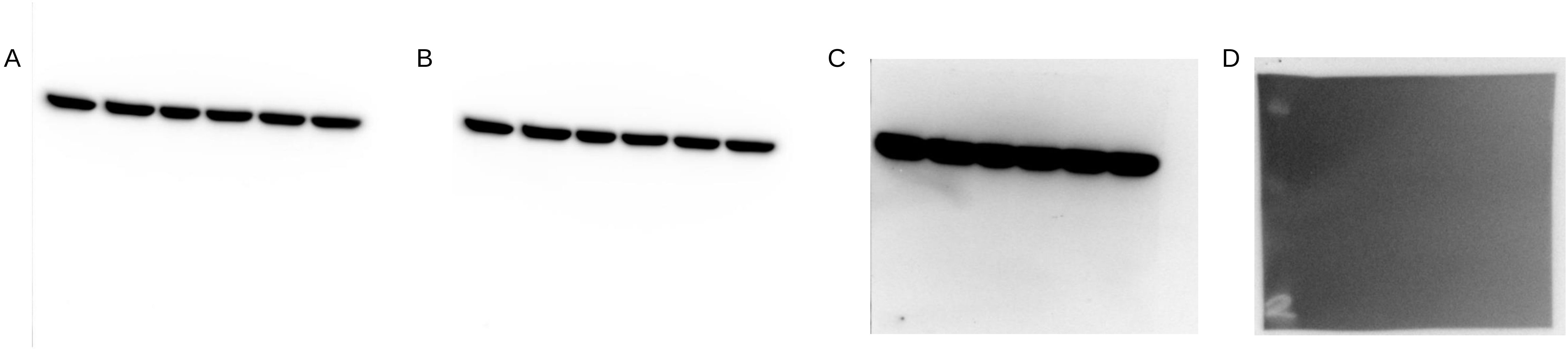

Supplementary Figure S9. Several exposure images of Actin Western Blot of Fig. 4. and imaged membrane. A) to C) several exposure images of Actin Western Blot of Fig 4. D) Image of the membrane from Actin Western blot of Fig. 4).
